# Temporomandibular joint arthritis in juvenile idiopathic arthritis, now what?

**DOI:** 10.1186/s12969-018-0244-y

**Published:** 2018-04-25

**Authors:** Matthew L. Stoll, Chung H. Kau, Peter D. Waite, Randy Q. Cron

**Affiliations:** 1 Department of Pediatrics, University of Alabama at Birmingham (UAB), 1600 7th Avenue South, Children’s Park Place North Suite G10, Birmingham, 35233 AL USA; 2 Department of Orthodontics, UAB, 1720 2nd Avenue South, School of Dentistry Building 305, Birmingham, 35294 AL USA; 3 Department of Oral and Maxillofacial Surgery, UAB, 1720 2nd Avenue South, School of Dentistry Building 419, Birmingham, 35294 AL USA

**Keywords:** Intraarticular corticosteroids, Juvenile idiopathic arthritis, Magnetic resonance imaging, Temporomandibular joint, Treatment

## Abstract

**Background:**

Arthritis involving the temporomandibular joint (TMJ) complicates 40 - 96% of cases of juvenile idiopathic arthritis (JIA), potentially leading to devastating changes to form and function. Optimal evaluation and management of this joint remains a matter of ongoing discussion.

**Methods:**

We performed a PubMed search for all articles with keywords “temporomandibular” and “arthritis”, covering the dates 2002 through February 28, 2018. A separate PubMed search was performed for all articles with keywords “temporomandibular joint”, “arthritis”, and “treatment” covering the same dates.

**Findings:**

The TMJ is a particularly challenging joint to assess, both clinically and with imaging studies. Clinical assessment of the TMJ is hampered by the low sensitivity of joint pain as well as the absence of physical exam findings early in the disease process. As with all joints, plain radiography and computed tomography only detect arthritic sequelae. Additionally, there is mixed data on the sensitivity of ultrasound, leaving magnetic resonance imaging (MRI) as the optimal diagnostic modality. However, several recent studies have shown that non-arthritic children can have subtle findings on MRI consistent with TMJ arthritis, such as joint effusion and contrast enhancement. Consequently, there has been an intense effort to identify features that can be used to differentiate mild TMJ arthritis from normal TMJs, such as the ratio of the enhancement within the TMJ itself compared to the enhancement in surrounding musculature. With respect to treatment of TMJ arthritis, there is minimal prospective data on medical therapy of this complicated joint. Retrospective studies have suggested that the response to medical therapy of the TMJ may lag behind that of other joints, prompting use of intraarticular (IA) therapy. Although most studies have shown short-term effectiveness of corticosteroids, the long-term safety of this therapy on local growth as well as on the development of IA heterotopic bone have prompted recommendations to limit use of IA corticosteroids. Severe TMJ disease from JIA can also be managed non-operatively with splints in a growing child, as well as with surgery.

**Conclusion:**

In this review, we summarize literature on the diagnosis and management of TMJ arthritis in JIA and suggest a diagnostic and therapeutic algorithm for children with refractory TMJ arthritis.

## Background

Forty to ninety-six percent of children with juvenile idiopathic arthritis (JIA) develop arthritis of the temporomandibular joint (TMJ) [1–6]; all JIA categories are at risk [7]. There are several features of this joint that warrant particular attention, including its importance for everyday function, potential cosmetic implications of altered dentofacial growth, and the challenges in the evaluation and management of TMJ arthritis. Detailed discussion of the functional implications of TMJ arthritis are available [8, 9], but briefly include pain with talking, difficulty eating, and obvious and potentially embarrassing alterations to the normal facial appearance. This review will focus on the diagnosis and management of TMJ arthritis in children with JIA.

## Methods

This was not a systematic review. However, one of the authors (RQC) performed a PubMed search for all articles with keywords “temporomandibular” and “arthritis”, covering the dates 2002 to the present. For the review of the studies on intraarticular therapy to the TMJ, a different author (MLS) performed a PubMed search for “temporomandibular joint”, “arthritis”, and “treatment” covering the same dates.

### Anatomy and function

The TMJ is a synovial joint composed of 4 articulating surfaces: glenoid fossa of the temporal bone, the upper and lower surfaces of the articular disc, and the mandibular condyle [10]. The disc divides the joint into the superior and inferior compartments. As it can move independently of the condyle, there is a potential for disc displacement, which results in pain, joint noises, and limited range of motion [11]. The TMJ is a complex joint termed ginglymoarthrodial, meaning that it has both hinge and sliding motion. Specifically, motion at the inferior compartment consists of rotation (ginglymoid joint) and manifests as moving the chin, while motion at the superior compartment consists of sliding or translation and manifests as protrusion of the mandible. Both movements are very important for maximum mouth opening and function [12]. A unique aspect of the joint is that both right and left must work in synchrony with partial dislocation. Additionally, the jaw works to maximize intercuspation of the teeth, so any dental anomalies can alter TMJ function and consequently result in condylar or disc abnormalities [11]. The fact that teeth create an abrupt stop and that malocclusion causes complex neuromuscular feedback with altered proprioception leads to a variety of symptoms, presenting as articular and myofascial pain and dysfunction.

### Evaluation of TMJ arthritis

#### History

The TMJ is among the more challenging joints to evaluate clinically, due to the absence of visible joint swelling and lack of symptomatology early during arthritis. Historical findings indicative of damaging TMJ arthritis include the usual symptoms of pain and stiffness, as well as TMJ-specific symptoms of clicking and popping. The former indicates irregularities of the disc with movement, while the latter indicates a sudden prominent movement or dislocation of the disc during translation [13]. A loud pop may indicate abnormal movement of the disc such as anterior dislocation with or without re-capture, limiting the range of motion. Joint noise is obvious due to close proximity to the ear cartilage and is commonly asymptomatic. The predictive power of such historical findings has been evaluated in studies of children with JIA, with findings that their sensitivities  are low. For example, Weiss et al. (2008) prospectively evaluated 32 newly diagnosed subjects with JIA, finding that symptoms of TMJ pain and dysfunction were only 26% sensitive, albeit 100% specific, for identification of TMJ arthritis, as assessed by MRI [14]. Thus, while certain abnormal physical exam findings are strongly suggestive of TMJ arthritis, their absence is not reassuring. The Weiss study, as well as similar studies evaluating physician examination maneuvers (below), used the MRI with contrast as a gold standard, the limitations of which will be discussed below.

#### Physical examination

TMJ arthritis does not typically manifest with joint swelling. Moreover, physical exam findings are late in the disease process where the bone growth has been altered by the arthritis. Thus, physical examination consists at the very least of evaluation for joint tenderness, clicking upon mouth opening, asymmetric mouth opening (present only in unilateral or unequal disease, with the jaw deviating towards the more affected side) [15], and assessment of opening. Recently, published recommendations also encouraged palpation of masticatory muscles and an evaluation of TMJ morphology and symmetry [16]. As with the historical signs, no single one of these markers is highly sensitive for arthritis. For example, Koos et al. (2014) prospectively evaluated five physical exam maneuvers (asymmetric mouth opening, pain on palpation of masticatory muscles, pain on palpation of the TMJ, TMJ clicking and reduced maximal incisal opening (MIO)) as predictors of TMJ arthritis, using MRI as the gold standard [17]. The sensitivity of each individual item ranged from a low of 21% (MIO) to a high of 65% (asymmetric opening). Combining the items, the presence of any one of them had a sensitivity of 85%, which will still not only miss a substantial number of cases but is also associated with a low specificity of 54%. Similarly, the studies by Weiss et al. (2008) and Muller et al. (2009) both reported that physical examination maneuvers had low sensitivity as well as low specificity for the detection of MRI-suggested TMJ arthritis in new-onset patients [14, 18]. In contrast, Abramowicz et al. (2013) reported that a combination of abnormal MIO for age and jaw deviation had a positive predictive value of 100% in patients with long-standing JIA, indicating that patients with both had a 100% likelihood of TMJ arthritis. However, in support of the previous work, the negative predictive value was only 46%, meaning that the majority of patients lacking one or both of these findings still had arthritis [19]. Kristensen et al. [19] performed a systematic literature review, concluding that while studies were not directly comparable, no single physical exam finding could accurately predict MRI findings of TMJ arthritis [20].

#### Plain radiography and computed tomography

As with any joint, radiography of the TMJ provides information only on arthritic sequelae, not active arthritis. The TMJ is difficult to image due to the overlay of the skull base especially by traditional films. Even standard panoramic tomograms contain artifact and are of little value compared to MRI and CT. Computed tomography (CT) provides greater anatomic detail as compared to plain radiography, and is thus of benefit primarily in identifying surgical candidates [21]. A form of CT, known as cone beam CT (CBCT), provides greater focus on the TMJ, thereby minimizing radiation of the surrounding brain and face. Features such as condylar flattening and erosion, as well as osteophyte formation, were readily distinguished between JIA patients and controls who underwent CBCT for unspecified reasons [22].

#### Ultrasound

Compared to MRI, ultrasound (US) has advantages with respect to cost and lack of requirement for sedation, but it is unclear as to whether it can identify active inflammation and arthritic sequelae as accurately as MRI with contrast. Weiss et al. (2008) compared US and MRI in the same cohort of 32 children studied above, finding that MRI detected both more active (24/32 vs 0/32) and chronic (22/32 vs 9/32) changes [14]. Likewise, Muller et al. reported that MRI and even physical examination were both more sensitive at the detection of active inflammatory changes and arthritic sequelae as compared to US [18]. More recently, Kirkhus et al. compared the correlation between ultrasonography-assessed capsular width and MRI assessment of synovitis (T1 weighted [T1W] signal increase at the synovium following administration of contrast), finding a correlation of 0.483 (*p* < 0.001) at the subcondylar level, concluding in contrast to the previous studies that US may in fact be a useful screening tool for arthritis of the TMJ [23]. In support, several other studies that did not constitute direct comparisons with MRI did show that US frequently detected findings of active arthritis in children with JIA [24–26]. The reason for the variation in these findings is not clear, although they may relate to the operator-dependence of US, as well as challenges to US due to the small anatomy of the TMJ of young children. A review of the literature concluded that US has low sensitivity for detecting joint effusion and may be more valuable to monitor established TMJ arthritis than for its initial detection [27].

#### Magnetic resonance imaging

**Fig. 1:**
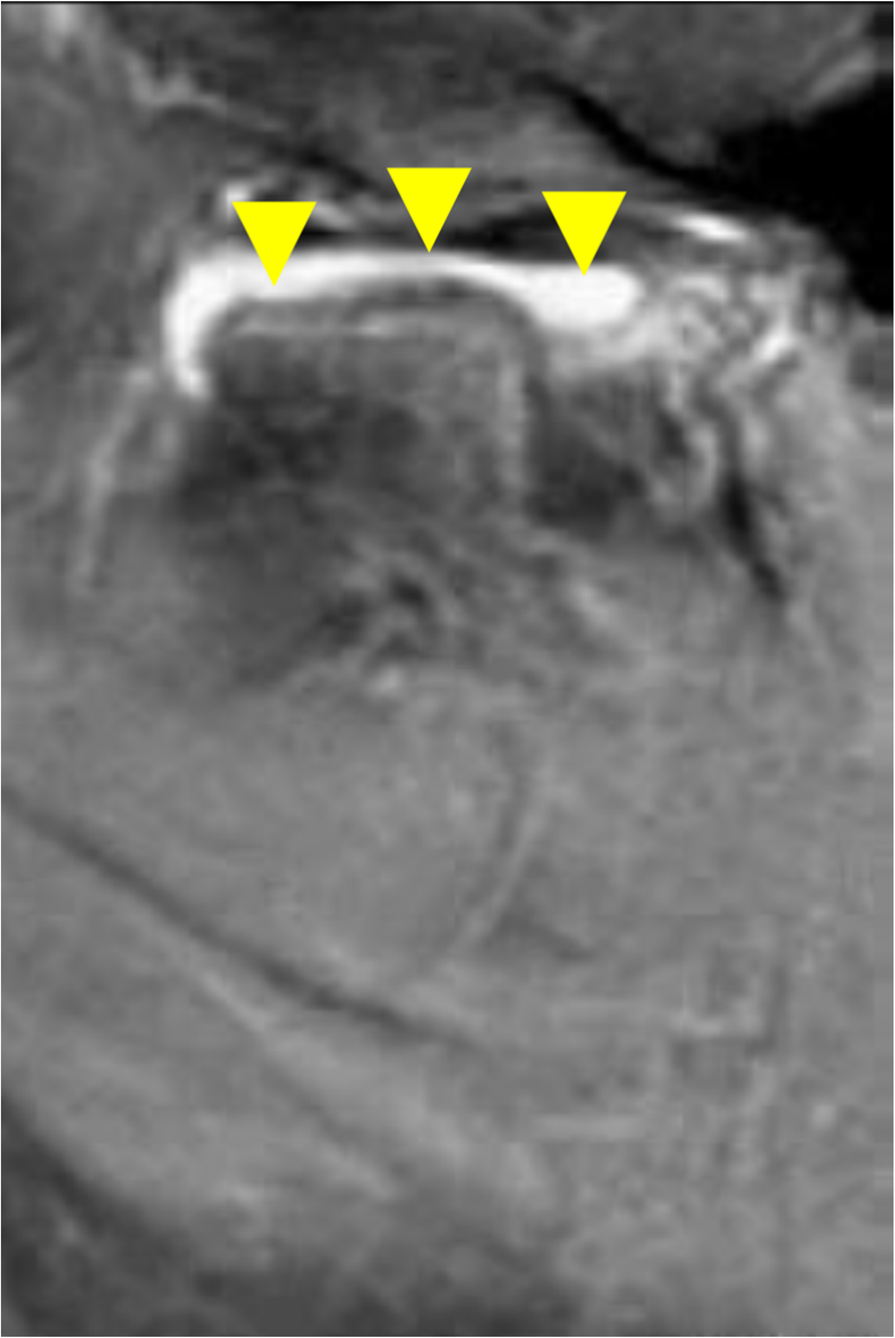
Active arthritis. Thickened synovium and contrast enhancement seen in the sagittal image of the left TMJ of a 13-year-old female with poly-articular JIA (arrowheads)

**Fig. 2:**
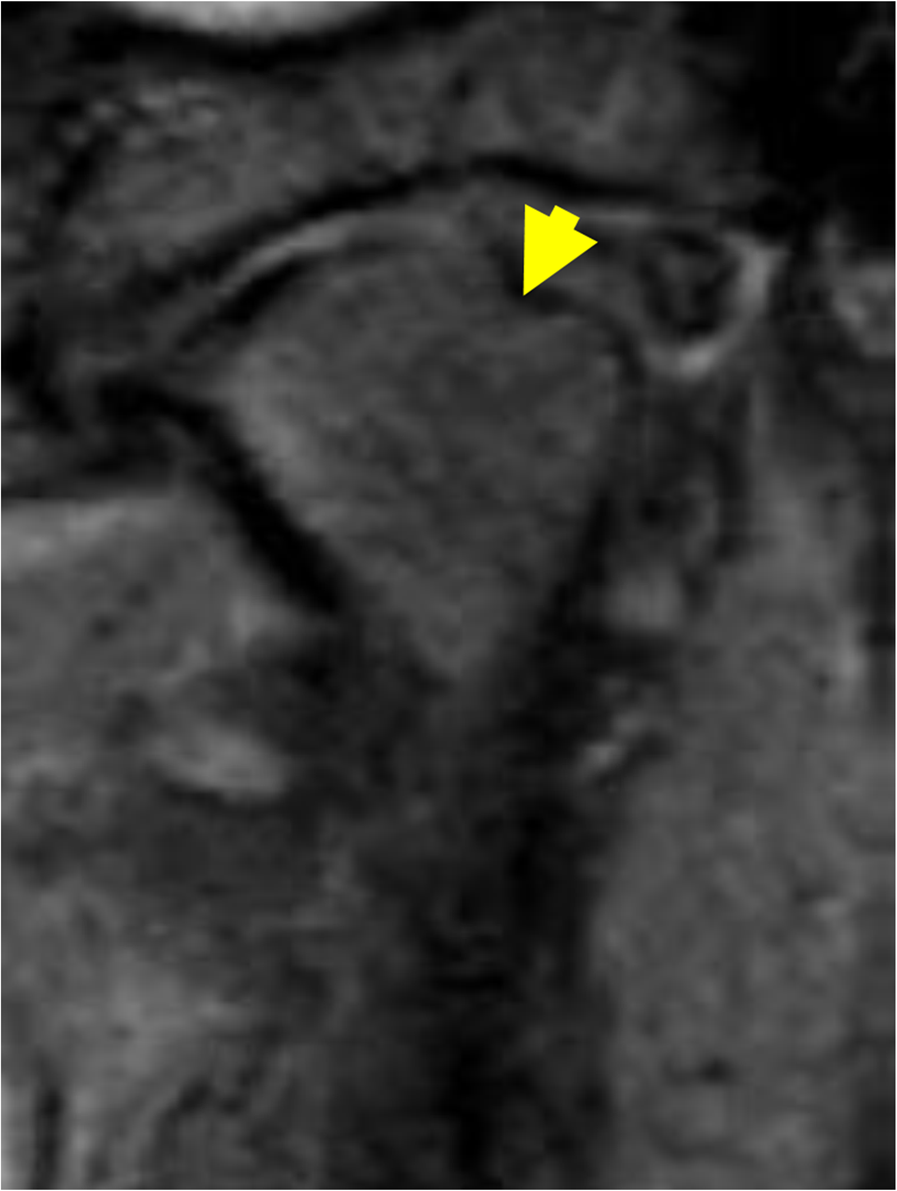
Arthritic sequelae. Large condylar erosion noted in the sagittal image of the right TMJ of an 11-year-old male with ERA/JIA (arrowhead)

**Fig. 3:**
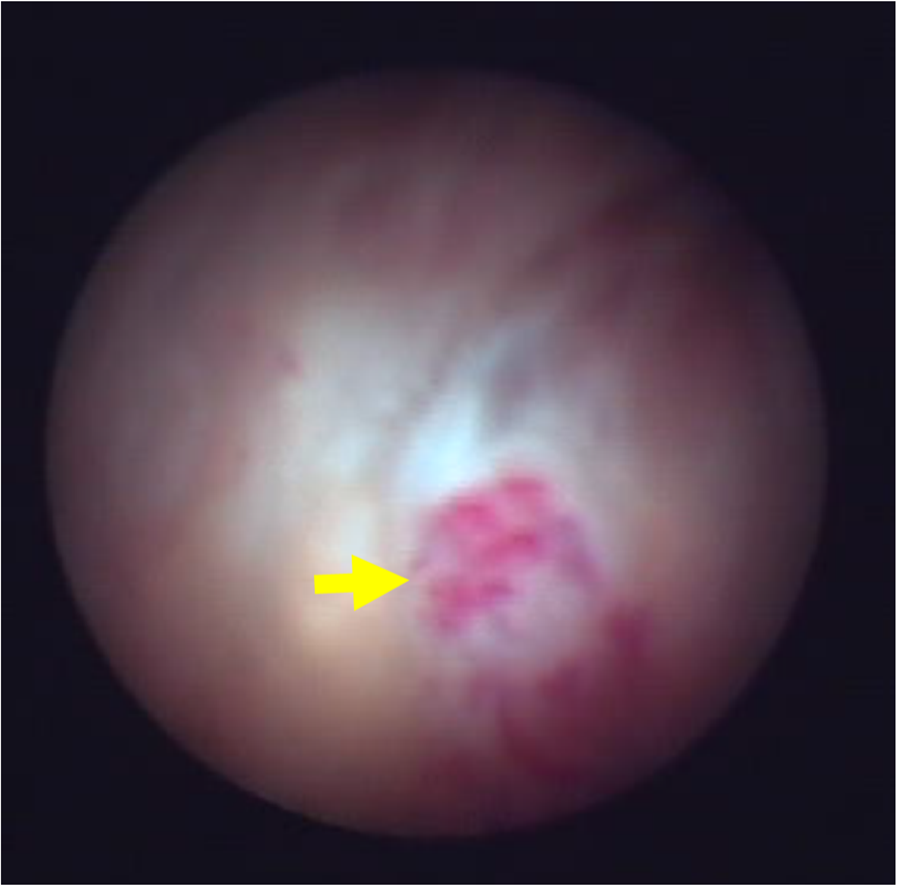
Arthroscopic images of the inside of a temporomandibular joint in a 17-year-old female with poly-articular JIA. A TMJ image using a mini arthroscope (1.2 mm) reveals clear regions of inflammation (arrow)

Most studies use MRI with contrast as the gold standard for the evaluation of TMJ arthritis [28], as it can identify both active arthritis changes as well as arthritic sequelae. Findings suggestive of active arthritis include joint fluid, bone marrow edema, and contrast enhancement (CE) (Fig. 1); those representing arthritic sequelae include changes to the shape of the condyle or disk, pannus, and osteophytes (Fig. 2). Short of performing biopsies or direct visualization (Fig. 3) of the joint in children with suspected TMJ arthritis, there would be no way to assess the sensitivity of the MRI in a human population. However, its specificity can be assessed by evaluating MRI of the TMJ in children who do not have arthritis. Although, ideally, such studies would be performed in completely healthy children, the requirement for CE, and in many cases sedation, preclude such a study for ethical reasons.

Nevertheless, several studies have evaluated findings at the TMJ in children without known or suspected JIA undergoing brain MRI. The first of these was conducted by Tzaribachev et al. in 2009; this retrospective study found that arthritic changes are very rare in non-arthritic children, with only three of 96 healthy children showing effusions and another three showing CE [29]. Unfortunately, multiple subsequent studies have shown contradictory findings. In an uncontrolled study, von Kalle et al. reported that 14 joints from 46 non-arthritic children undergoing MRI of the brain had some degree of CE; additionally, the intensity of CE in the joint tissue post-contrast was 73% higher than pre-contrast, while the intensity of the CE in the joint tissue was a more modest 20% higher than that of the surrounding musculature [30]. An even higher frequency of CE was reported by Kottke et al. in their study of 27 non-arthritic children [31]. Fifty-two of 54 TMJs (96%) demonstrated CE, and 43 of 54 (83%) had effusions. Likewise, Angenete et al. reported CE in 35 of 36 (97%) subjects, as well as mild flattening of the condyle in 20/101 [32]. Finally, Stoll et al. [33] reported CE in 120/122 (98%) non-arthritic controls, with the average size of the enhancement actually larger as compared to that in 35 newly-diagnosed JIA patients (1.1 ± 0.24 vs 0.88 ± 0.27 mm, *p* < 0.001).

In addition to identifying the presence of joint fluid or enhancement in non-arthritic subjects, several recent studies have also sought to determine the optimal method of assessing the presence and extent of enhancement. As CE will increase in any tissue with passage of time following contrast administration [34], comparison of CE in the TMJ with that of a control site present within the field of the view, most commonly the longus capitus muscle, is one approach. This ratio of the signal intensity (SI) in the area of interest divided by the SI in a control location is generally referred to as the enhancement ratio (ER). This method was studied by Peacock et al. in their study of 158 non-arthritic children who underwent MRI of the head. They reported ERs of 1.52 and 1.68 for the inferior and superior joint spaces, respectively [35]. The same group also performed a retrospective controlled study of 72 children with JIA and 71 non-arthritic controls. In this study, both JIA patients and controls had an ER greater than 1, while the JIA patients had a significantly higher ER as compared to the controls (2.52 ± 0.79 versus 1.28 ± 0.16), with ROC analysis identifying 1.55 as the best cutoff value [36].

Similarly, Caruso et al. evaluated three different ratios in a cohort of subjects that included JIA patients with symptoms suggestive of TMJ arthritis, JIA patients without such symptoms, and non-arthritic controls. These ratios were (postGadolinium SI in the TMJ – preGadolinium SI in the TMJ)/(postGadolinium SI in the longus capitus – preGadolinium SI in the longus capitus); (postGadolinium SI in the TMJ – preGadolinium SI in the TMJ)/(postGadolinium of longus capitus); and (postGadolinium SI of TMJ)/(postGadolinium SI of longus capitus). Of those three, they concluded that the most favorable measure was the second (postGadolinium SI in the TMJ – preGadolinium SI in the TMJ)/(postGadolinium of longus capitus), due to optimal discrimination among the three groups and a lack of a substantial increase over time [37]. Likewise, Ma et al. [38] compared a metric that evaluated only the change in SI pre- versus post-administration of gadolinium with a metric that measured a signal to noise ratio based upon enhancement in surrounding tissue, studying 67 children with JIA and 24 non-arthritic controls. Consistent with the study by Caruso et al. [37], Ma et al. concluded that the ER, which incorporated the extent of enhancement in the surrounding tissue, was better able to discriminate JIA patients with mild disease from controls [38]. While the optimal method of assessing the extent of TMJ joint fluid or enhancement in controls may not be entirely clear, it is evident that small amounts of joint fluid and CE in non-arthritic subjects are common. Lastly, there also remain questions as to the optimal protocols, and magnet strengths versus imaging coils in evaluating the TMJ by MRI in children with JIA [39, 40].

Findings of mild degrees of CE in non-arthritic children should not undermine the body of literature indicating a very high frequency of TMJ arthritis in children with JIA. Healthy children do not typically demonstrate retrognathia, micrognathia, and jaw deviation on exam, findings that were once the norm in children with JIA [41–43]. Finally, all of these recent studies of the MRI in non-arthritic children reported fairly mild active arthritic changes, and essentially absent arthritic sequelae, a clear distinction from that seen in children with JIA [14, 41].

### Medical treatment of TMJ arthritis

There is minimal prospective data evaluating the effectiveness of systemic immunosuppressive therapy on TMJ arthritis. Randomized clinical trials of conventional and biologic disease-modifying anti-rheumatic drugs generally have not included the TMJ as an outcome. The only prospective study that did evaluate the effectiveness of systemic medications on the TMJ was published over 30 years ago and included two medications that are no longer used (gold and penicillamine) in the management of children with JIA or related disorders [44]. Evidence that the TMJ might not respond as well to current therapies as compared to other joints is observational, e.g., a retrospective study showing that of 73 patients with no evidence of arthritis on physical exam; 36 (49%) nevertheless had TMJ arthritis detectable by MRI [6]. Many of these patients were taking traditional and biologic disease-modifying anti-rheumatic drugs (DMARDs). It is unclear why the TMJ would respond less robustly to systemic medications, as compared to other joints. The joint space is physically close to the growth zone of the condylar head, and evolutionarily it is a distinct synovial joint with a unique biochemical makeup. Moreover, there is precedent for the observation of relative responsiveness to therapy with other joints, e.g., the inability of traditional DMARDs to treat axial spondyloarthritis despite some effectiveness with these medications in the management of peripheral disease [45]. There may also be differences in the biology of arthritis in that joint; as an illustration, one study showed different epigenetic changes of fibroblast-like synoviocytes in the knee as compared to the hip of patients with rheumatoid arthritis [46].

Despite these observations, there is indirect evidence that the TMJ does in fact respond to systemic immunosuppressive therapy. As discussed above, progressive radiographically evident destructive changes were once the norm in children with JIA, while this no longer appears to be the case. Anecdotally, our clinics are no longer heavily populated with children with visually evident facial deformities. Data supporting these observations came from a study by Twilt et al., who performed baseline and 5-year radiographs in 70 children treated with systemic but not local immunosuppressive therapy, finding decreased evidence of TMJ changes on exam as well as by radiography [47]. The findings are all the more impressive given the minimal usage of biologics in this cohort (6/70; Twilt, personal communication). Likewise, Ince et al. reported decreased radiographic evidence of TMJ arthritis among 18 patients with JIA who were taking methotrexate, compared with nine who were not [48]; this was not a controlled study, so it is likely that the children on methotrexate were perceived to have had more severe disease overall than the children not taking any therapies, thus potentially biasing the findings towards the opposite direction. Finally, Stoll et al. (2012) reported that disease duration was protective against the likelihood of having TMJ arthritis in a population of 187 children with JIA, a finding which they took to indicate that therapy itself was protective [6]. These findings are clearly in stark contrast to the older literature, in which disease duration was associated with progressive radiographically evident deterioration [42].

### Intraarticular therapy for TMJ arthritis

The safety and effectiveness of intraarticular corticosteroid injections (IACI) for TMJ arthritis has been reviewed twice, with somewhat different conclusions despite inclusion of the same studies [49, 50]. Included studies, as well as studies published since these reviews, are summarized in Tables 1 and 2. These studies have generally reported short-term improvement in markers of TMJ arthritis, including pain, physical examination findings, and MRI findings. Moreover, results were more robust in some studies versus others. In addition, no short-term serious adverse events were reported therein. As Stoustrup (2013) reported, these studies, however, generally lack methodologic rigor, as they are retrospective, uncontrolled, and unblinded in the outcome assessments, among other limitations [50]. In addition, the studies may not have captured one recently identified potential safety event: alterations in the growth potential at the TMJ. Lochbuhler et al. performed IACI in 33 children with JIA, finding impaired mandibular growth following this therapy [51]. A unique aspect of this study was that the investigators performed MRI at the time of the injection to evaluate whether the corticosteroid was administered within or immediately outside the joint space. Those subjects who received successful IA placement of the drug demonstrated decreased grade of inflammation yet more impairment of mandibular growth as compared to those subjects in whom MRI demonstrated extra-articular placement of the corticosteroid. Additionally, 21% of the subjects developed heterotopic bone formation (HBF) in the TMJ, which the authors speculated might have resulted from the CS injections themselves, and higher cumulative corticosteroid doses were associated with increased risk of condylar damage, although the issue of confounding by indication was not addressed. Nevertheless, the possibility that IA therapy could promote HBF was subsequently corroborated by Stoll et al. in their study of 238 subjects who had received IACS therapy, of whom 33 developed this outcome; in this study, multivariable analysis revealed that the total number of injections was associated with increased risk of HBF, while delay from diagnosis of JIA to initial injection was protective [52]. Finally, one additional limitation of the studies evaluating the effectiveness and safety of IACI into the TMJ is that as they were all relatively small, they may not have captured rare but potentially serious short-term SAEs, such as rapid TMJ destruction and ankylosis [53, 54].

**Table 1:** Overview of studies evaluating local therapy for TMJ arthritis

Study	*n*	Therapy	Injections/TMJ	Localization of IACI	Duration of follow-up
Arabshahi et al. [122]	23	TA 40 mg; TH 20 mg	1	CT	6–12 months
Ringold et al. [106]	25	TA 20–40 mg; TH 10–20 mg	1–5	Anatomic	26 months (5–52)
Weiss et al. [14]	21	TH 10 mg	1	CT	6 months
Parra et al. [123]	83	TH 5–10 mg TA 5–10 mg	1–6	US	6 weeks
Mina et al. [67]	28	DIP 6 mg	8–10	Anatomic	Completion of course
Habibi et al. [124]	39	TH 10–20 mg	1	US	6–8 weeks
Stoll et al. [125]	63	TH 5–10 mg	1–2	Anatomic	5 months
Stoll et al. [56]	24	INX 5–10 mg	ND	Anatomic	7.8 months
Olsen-Bergem et al. [64]	21	Arthrocentesis plus Triamcinolone	1	US	8 months
Olsen-Bergem et al. [64]	17	Arthrocentesis alone	1	US	8 months
Lochbuhler et al. [51]	33	TH 6–20 mg	1–7	Anatomic	5 years
Stoll et al. [55]^1^	33	INX	1–7	Anatomic	9 months (2–27)
Stoustrup et al. [126]	13	TH 20 mg	1	Anatomic	333 days (190–600)
Kinard et al. [65]	3	Arthrocentesis alone	1	Anatomic	1 month
Resnick et al. [127]	29	TH 10 mg	1	Anatomic	22.9 months
Resnick et al. [128]	45	TH 10 mg	1	Anatomic or imaging^2^	21–22 months
Antonarakis et al. [66]^3^	21 (IACS), 8 (lavage)	TA 20 mg	1	Anatomic	6 months

^1^There is overlap in patients with Stoll et al. [56]; however, patients present in both studied had undergone additional injections in the intervening period. ^2^This study compared patients who had received injections via anatomic guidance versus those who had received imaging guidance. For the latter, multiple modalities (CT, US, fluoroscopy) were used. ^3^This study compared TMJ lavage alone with lavage plus IACI. *Abbreviations*: *DIP* demamethasone iontophoresis, *INX* infliximab, *TA* triamcinolone acetonide, *TH* triamcinolide hexacetonide

**Table 2:** Outcome of studies evaluating local therapy for TMJ arthritis

Study	Subjective change	Physical exam change	Imaging change	Safety
Arabshahi et al. [122]	Resolution of pain in 10/13 subjects	MIO increase of 4.8 mm	Improved active findings on MRI in > 67% of TMJs (14 subjects)	Transient Cushing syndrome in 2 subjects
Ringold et al. [106]	Decreased incidence of one or more TMJ symptoms (60% to 28%)	MIO increase of 6.6 mm; decreased incidence of jaw deviation (40% to 16%)	CT: worsening changes in 10, no change in 3, and improvement in 2 subjects	Subcutaneous atrophy in 1 subject, IA calcification in two subjects
Weiss et al. [14]	ND	Improved MIO in 9/16 abnormal at baseline	Decreased MRI findings of active arthritis in 5/6	ND
Parra et al. [123]^1^	“Good” response in 80/99 encounters, “Partial” response in 10, and “Poor” response in 9	ND	ND	Skin atrophy in 1 subject
Mino et al. [67]	Resolution of pain in 11/15 (73%) with pain at baseline	Improved MIO of 5 mm among the 18 patients with decreased MIO at baseline	ND	Transient painless erythema in 24/28 (86%); metallic taste in one subject
Habibi et al. [124]	Improved pain in 17/17 subjects and improved chewing dysfunction in 5/7 subjects	Improved jaw deviation in 13/14 subjects	ND	Scar in one subject
Stoll et al. [125]	ND	Increased MIO by 2.7 mm	Of 62 TMJs: 24 improved, 30 stable, 8 worse	One subject each with localized swelling, fever x two weeks, and hypopigmentation
Stoll et al. [56]	ND	No change in MIO	No improvement overall by MRI; resolution of inflammation in six TMJs	No AEs
Olsen-Bergem et al. [64]	Improved pain at rest and with palpation	Increased lateral excursion of 3.7 mm (Triamcinolone group)	ND	ND
Olsen-Bergem et al. [64]	Improved pain at rest and with palpation	Increased lateral excursion of 4.6 mm (arthrocentesis alone group)	ND	ND
Lochbuhler et al. [52]^2^	ND	ND	Improved inflammatory grade of MRI	Decreased growth of mandibular ramus
Stoll et al. [55]	ND	No change in MIO	Worsening of active and chronic MRI findings	ND
Stoustrup et al. [126]	Improved short-term pain frequency and intensity	No significant changes in MIO, laterotrusion, or protrusion	ND	ND
Kinard et al. [65]	Decreased pain	Improved MIO	ND	Transient subcutaneous atrophy
Resnick et al. [127]	Decreased pain	Improved MIO of 5.8 mm	Decreased ER of 1.06	ND
Resnick et al. [128]	Resolution of pain in 34/37 (92%)	Improved MIO of 5.0 mm (anatomic) or 5.1 mm (image)	Decreased ER of 1.16 (anatomic) or 0.96 (image)	ND
Antonarakis et al. [66]	TA: Decreased VAS 2.6 L: Decreased VAS 1	TA: Improved MIO of 2.3 mm L: Improved MIO of 1.4 mm	TA: Improved in 18 / 42 TMJs L: Improved in 5/16	ND

^1^Some of the reports reflect children who had more than one round of injections. ^2^Intra-articular placement was evaluated with MRI. Those with IA placement demonstrated more robust improvement but more impairment of mandibular growth. *Abbreviations*: *ER* enhancement ratio, *L* lavage alone, *MIO* maximal incisal opening, *ND* not documented, *TA* triamcinolone acetonide, *TMJ* temporomandibular joint

Another form of IA therapy that has been proposed is IA infliximab [55, 56]. IA therapy with tumor necrosis factor inhibitors into large joints has shown some effectiveness, e.g. [57, 58], even among patients who have failed IACI [59], and may be equivalent to if not superior than some forms of IA corticosteroids [60–62]. There is a single case report of 8 IA injections of infliximab administered to the TMJ over 36 weeks in an adult patient with psoriatic arthritis who had previously failed therapy with systemic infliximab as well as local IACI [63]. This patient had clinical improvement without radiographic deterioration; MRI was not used as an outcome measure. Unfortunately, studies in children with JIA have not been able to replicate this success [55]. The dose that can be administered into the TMJ of a child may be a limiting factor; the study by Carubbi et al. (2016) demonstrated superiority of TNFi over CS only in large joints [62]. Additionally, the subject selection of JIA patients refractory to traditional and biologic DMARDs plus IA CS is one that is not ideal for the assessment of the effectiveness of IA infliximab. As subsets of TMJ arthritis patients anecdotally appear to benefit from IA corticosteroids and IA infliximab, both in the short and long terms, it will be valuable to identify the factors associated with likelihood of response to these therapies.

Finally, arthrocentesis and lavage without injection of any therapies may also have anti-inflammatory effects. Olsen-Bergem et al. randomized 17 JIA patients with bilateral TMJ arthritis to receive arthrocentesis alone in one joint, versus arthrocentesis accompanied by triamcinolone hexacetonide (TH) in the other; an additional four subjects presumably with unilateral involvement received TH plus arthrocentesis unilaterally, somewhat compromising the analysis of the study [64]. The authors reported improvement in subjective parameters and objective physical examination findings in both groups compared to baseline, without any evident differences between the two treatment groups. Likewise, Kinard et al. published a case series of three children with JIA who underwent arthroscopy with lavage alone, reporting decreased pain at one-month follow-up in all three [65]. Improvements in MIO of 2 and 5 mm were reported in two subjects; a third had unspecified improvement. Most recently, Antonarakis et al. compared outcomes of children receiving IACI with lavage, lavage alone, and no therapy [66]. There appears to have been non-random assignment to all three groups, and they indicated that some of the children who received IACI to one TMJ may have received lavage in the contralateral joint, thus compromising assessment of change in MIO. They reported improvements in both treatment groups, perhaps more so in the group that also received IACI, but few differences that were statistically significant as compared to the no-treatment group. Thus, the benefit of lavage alone remains an open question. Additionally, despite short-term success of IA therapy in general, long-term benefit of IA therapy remains in question.

### Iontophoresis

An alternative method of delivering CS to the TMJ was introduced by Mina et al. [67]. This procedure consists of transdermal application of the drug, which is forced into deeper tissues through application of an electrical current. It has been used sporadically in arthritis [68, 69]. Their results were promising, with improved MIO observed in 19/28 and decreased pain observed in 11/15 with pain at baseline. Factors that may limit widespread application of this technique are that this requires a trained physical therapist to perform, as well as multiple visits to their office. No additional studies of this approach in treating TMJ arthritis have been reported.

### Orthodontic (functional) devices

In order to preserve normal facial and jaw growth, mechanical (non-anti-inflammatory) approaches have been used in children with JIA. Functional orthodontic appliances (FOA) are splints that can alter mandibular position by stretching local musculature [70], basically braces for the jaw. Two types of FOA are available,: active treatment and distraction (stabilization) splints [70]. Occlusal stabilization splints are used to help support and balance both TMJs and to prevent further pain and discomfort to the TMJ complex. They can be used in growing as well as in skeletally mature patients. They allow the patient to have even contacts when the teeth occlude in all ranges of motion including biting and side to side jaw movements, which can result in decreased pain [71]. In contrast, active treatment splints are only used in the growing phases of a child, typically ages 8–16 years of age, and are intended to add incremental height to the splint platform on the affected side of the arthritic joint, thus potentially reducing asymmetry and need for surgical correction of skeletal deformity [72]. They can also result in more even distribution of muscular forces within the jaw. Both forms of FOA are fairly unobtrusive cosmetically but effective therapy often requires many years of compliance. The general consensus is that they are optimally used when the disease is well-controlled medically [70], although studies evaluating outcomes of JIA patients with versus without active TMJ arthritis who are treated with FOA have not been performed. As reviewed [7], there are no high-quality prospective studies on their effectiveness. Instead, data are generally limited to one large study of children with impaired jaw shape for a variety of reasons [73] and smaller studies limited to children with JIA [74–76], all of which appear to show modest benefit. A recently published retrospective study of 54 children with JIA who were treated with a FOA for two years demonstrated decreased pain and increased MIO, although there was no comparator group. Data in a rabbit model of induced TMJ arthritis demonstrated that stabilization splints significantly reduced the condylar destruction and bone loss compared to untreated rabbits with TMJ arthritis [77], providing rationale for prospective studies in children with TMJ arthritis. No major safety issues have been raised with these devices [70].

### Surgery

Once a child has reached skeletal maturity, surgery is the only means of correcting anatomic abnormalities. The consensus is that surgery is not optimally performed in clinically active TMJs, and is generally postponed until growth is complete [78]. However, if TMJ ankyloses develops, surgical intervention such as arthroplasty, or total prosthetic joint replacement is indicated sooner. Surgical options were reviewed in depth by Norholt et al. [78]. Briefly, two options are available: distraction osteogenesis and orthognathic surgery. The former is a procedure, in which a partial osteotomy is performed in the cortex of the ramus, and slow mechanical forces are created daily increasing the desired length. New bone is slowly generated similar to growth. This technique is commonly used in craniofacial deformities such as Pierre Robin Sequence with airway obstruction [79]. Orthognathic surgery is a common procedure to reconstruct the dento-skeletal deformity with precise masticatory function, and TMJ articulation, usually performed in young adults. This may involve a bilateral sagittal osteotomy of the ramus and/or Lefort 1 of the maxilla for alignment of the masticatory system with proper plane of occlusion to the TMJ [80].

### Experimental/future therapies

Several IA therapies have been attempted in animal models of TMJ arthritis, whose future applications to human disease remain uncertain. Most of these studies use a model in which disease is introduced in rats or rabbits through intra-TMJ injection of a compound called Complete Freund’s Adjuvant, which consists of heat-killed *Mycobacterium tuberculosis* and induces a robust immunologic response. Two groups evaluated low-level laser therapy (LLLT), showing improved histologic features of inflammation [81, 82]. Human studies of LLLT show that it may have a modest analgesic effect [83]; however, its potential mechanism in arthritis is uncertain, and its clinical effects in patients with rheumatoid arthritis appear modest [84]. Another potential therapy is local injection of hyaluronic acid (HA), which reduced histologic and bony morphometric measures of TMJ inflammation in one rat study [85]. There is extensive clinical experience with HA as a therapeutic agent for osteoarthritis OA, for which there is an FDA-approved indication [86]. There is also limited, although positive, experience with HA therapy in RA [87] and isolated enthesopathies [88]. Finally, one group treated juvenile rabbits with induced TMJ arthritis with IA simvastatin, reporting improved bone surface density, although the extent of inflammation was not assessed [89]. In addition to its cholesterol-lowering effects, statins may also have immunomodulatory properties, as evidenced by in vitro studies showing direct effects on the induction of regulatory T cells [90] and in vivo studies demonstrating modest but statistically significant improved disease scores in a randomized trial of atorvastatin versus placebo in adults with RA [91], and decreased risk of RA among long-term users of statins [92]. The potential role of any of these therapies in the management of TMJ arthritis in children with JIA remains unknown and speculative.

### Our approach to refractory or isolated TMJ arthritis in children with JIA

In a child presenting with possible isolated TMJ arthritis, the first step is to distinguish JIA limited to the TMJs from its mimic, idiopathic condylar resorption, alternatively called internal condylar resorption (ICR) [93]. A discussion of the surgical treatment of ICR is beyond the scope of this review, but is available elsewhere [94]. Like any other joint, the TMJ can be the initial or sole manifestation of JIA. Indeed, some children presenting with isolated TMJ arthritis will go on to develop arthritis in other joints or uveitis [95]. Differentiating isolated TMJ arthritis from ICR can be challenging, particularly in light of the data summarized above showing that non-arthritic children can have some degree of joint fluid or enhancement, so the presence of these findings, if mild, is not necessarily diagnostic of arthritis. Marked inflammatory changes, such as synovial thickening, appear to be rare in ICR [96], so when present, may suggest JIA. Erosive condylar changes may also help distinguish between the presence of ICR and JIA [22, 97]. In addition, while disc displacement in common in ICR, significant damage to the disc is rare [98]. Finally, unilateral involvement may also suggest JIA over ICR [99, 100], although this has not been established.

**Fig. 4:**
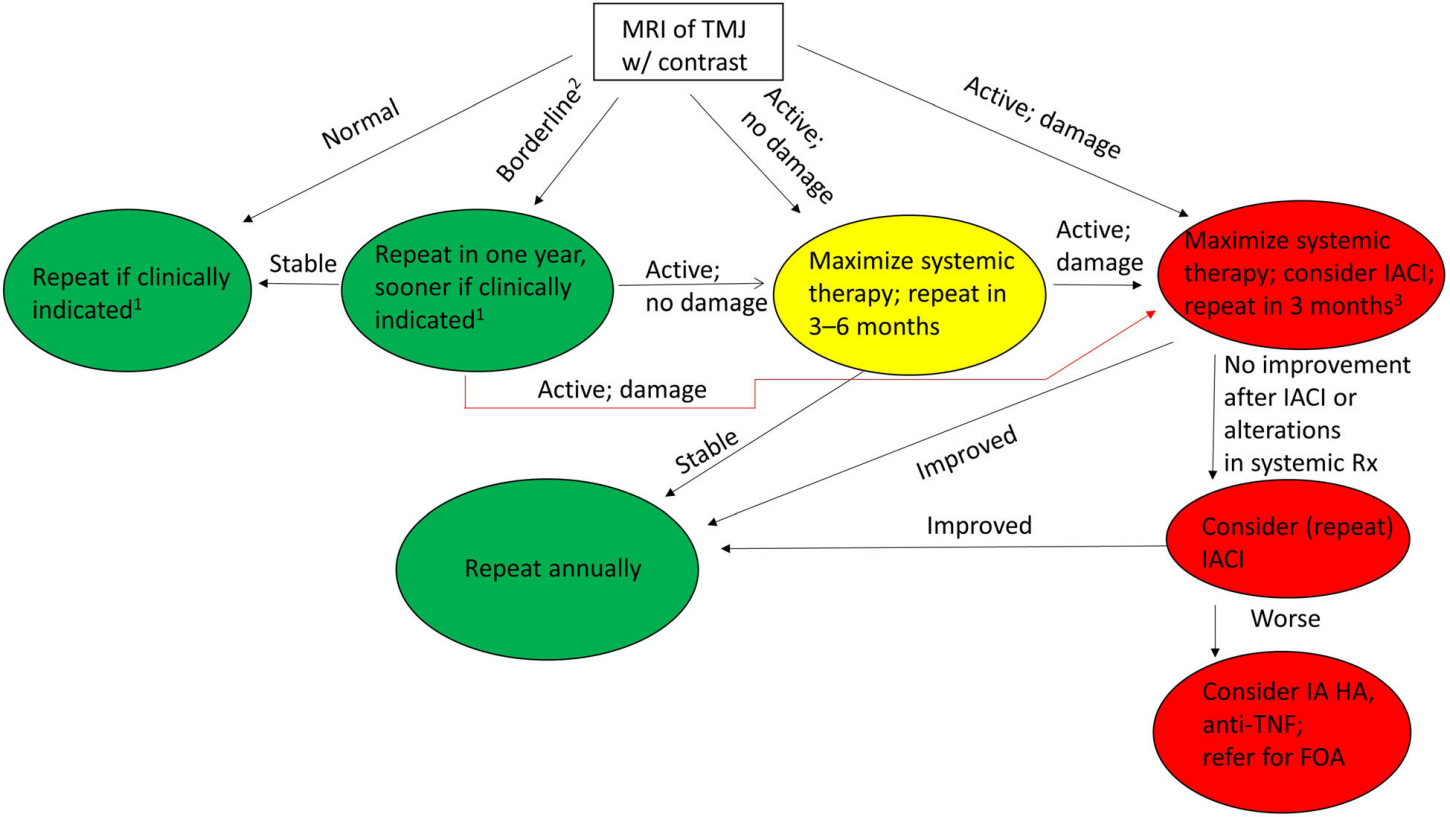
Recommended therapeutic approach to isolated TMJ arthritis. ^1^Develops new or worsening suggestive symptoms or physical exam findings. ^2^Mild active arthritis, similar to what can be seen in controls. ^3^If injected, then repeat MRI three months after injection. Otherwise, repeat 3 months after change in therapy. *Abbreviations*: *FOA* = functional orthodontic appliance. These recommendations reflect the opinions of the authors alone

A vexing scenario for the pediatric rheumatologist is a child with isolated TMJ arthritis, either at onset or following successful systemic therapy of the remainder of the joints [7]. The management will depend on a variety of factors, including extent of active arthritis and arthritic sequelae on imaging, presence of symptoms or exam findings associated with TMJ arthritis, and availability of corticosteroid preparations. (At the time of this writing, TH, which is the optimal corticosteroid preparation for IA therapy in JIA [101], is not available anywhere in the United States). A flow diagram is shown in Fig. 4.

Children with JIA and completely normal findings on the MRI of the TMJ generally do not warrant further investigations unless signs or symptoms of TMJ arthritis develop. In children with JIA who are old enough to cooperate with the exam, we will typically follow MIO measurements (measured with disposable TheraBite scales, Atos Medical, New Berlin, WI), longitudinally. Unless very low, a single measurement has little prognostic value due to the wide range of measurements in healthy children [102]. However, decreased MIO is likely to indicate TMJ arthritis, as is development of facial asymmetry and other signs or symptoms discussed above. Importantly, the smallest detectable difference in MIO was reported to be just under 0.5 cm [103], so changes of a lesser magnitude may not be clinically significant. Recently, recommendations for monitoring TMJ involvement in JIA were published [16].

In children with mild findings of active arthritis, e.g. effusions or areas of enhancement < 1.5 mm with or without mild bone marrow edema, we recommend repeating the MRI within one year, as these can be normal findings. These mild changes can be observed in non-arthritic pediatric subjects [33], but this does not necessarily mean that it is a negative study. As discussed above, cross-sectional studies using plain radiography clearly demonstrate TMJ changes in at least 40% of JIA patients [42], so the pre-test probability of TMJ arthritis is considerably higher in a JIA patient than in a non-arthritic control. If the findings do not progress over one year, then subsequent imaging studies may not be required.

Children with fairly extensive active findings clearly have TMJ arthritis associated with JIA. However, in light of the recently recognized risks associated with IACI in the TMJ on long-term growth of the joint, as well as risks of HBF, optimal management is uncertain. Such children should have their systemic therapy optimized, e.g., addition of a conventional or biologic DMARD or change in dose; specifically, uses of weekly adalimumab or infliximab at doses upwards of 10 mg/kg/dose have been reported as safe and effective in children with JIA [104, 105], and it may be reasonable to consider to consider switching biologics in some cases. Furthermore, these children should be followed closely for development of TMJ damage as evidenced by MRI and MIO, as well as assessments of dentofacial growth and development of asymmetry. If the arthritis is asymptomatic and is not damaging the joint, then adjustment of the systemic therapies and careful monitoring may be all that is required. However, if the TMJ arthritis is causing significant damage to the joint, e.g., bony erosions or disk displacement, then local therapy in addition to adjustment of systemic therapies may be recommended. It bears emphasis that while mild active changes can be seen in non-arthritic children, significant arthritic sequelae continue to be specific for arthritis, and the presence of such in the context of large areas of enhancement or thickened synovium therefore represent unopposed arthritis and undoubtedly place the child at risk of structural and functional damage. If these steps are not successful, we would not recommend performing more than two IACI into the same TMJ, as children who do not respond to an initial injection generally do not respond well to subsequent injections either [106]. If the arthritis is progressing despite these measures, then alternative albeit somewhat experimental approaches such as injection with a TNFi or HA, or even lavage alone, may be warranted. In addition, orthodontic approaches may be of value in maintaining appropriate jaw growth [71, 74, 107].

For management of TMJ arthritis, as with management of JIA as a whole, there is no clear guidance from the literature as to when therapies may be discontinued. Use of S100 proteins have been studied as a predictive tool among children discontinuing TNFi therapy [108], but these markers are not available for clinical purposes in the United States, nor is there any specific data with respect to their use in the TMJ. We would recommend that all other aspects of the disease (arthritis in other joints, uveitis, systemic symptoms, etc) should be in remission [109] for at least 12–24 months [110], although there is mixed data as to whether prolonged periods of remission increase success of drug withdrawal [111–114]. Then, if MRI with contrast reveals no active findings in the TMJ, one may consider tapering systemic therapy.

Finally, monitoring TMJ arthritis by contrast MRI has been questioned in terms of safety. In 2017, the Food and Drug Administration issued a statement calling into question the safety of gadolinium-based contrast agents (GBCAs; https://www.fda.gov/Safety/MedWatch/SafetyInformation/SafetyAlertsforHumanMedicalProducts/ucm559709.htm). This recommendation is based upon findings of retention of GBCAs in the brain and possibly other tissues following repeat studies [115]. It bears emphasis, however, that there no clear clinical symptoms associated with this deposition, and GBCAs have been safely used in millions of patients with normal renal function [116], and a revised statement released by the FDA in December of 2017 concluded that while we should minimize closely-spaced repeat contrast MRIs, we should not avoid or defer necessary scans (https://www.fda.gov/Safety/MedWatch/SafetyInformation/SafetyAlertsforHumanMedicalProducts/ucm589580.htm). It is advisable when possible to use macrocyclic rather than linear GBCAs, as the former result in decreased deposition [117, 118].

The issue of retention of GBCAs is unrelated to long-recognized safety issue with GBCAs: the risk of nephrogenic systemic fibrosis in patients with renal insufficiency [119]. In these patients, the risks and benefits of a contrast MRI must be weighed very carefully, and our general recommendations above do not apply to them.

## Conclusion

Once dubbed the “forgotten joint” [120], there has been an explosion of scholarship in recent years focusing on the diagnosis and management of TMJ arthritis. Yet, the more we learn about this joint, the less we really know about it. There is no doubt that TMJ arthritis is a frequent complication of JIA, and that if untreated, can have devastating effects on the form and function of the joint, jaw, and midface. While distinguishing between normal findings and mild arthritis can be challenging, significant TMJ arthritis resulting in joint damage can still occur, even early in the disease course [14]. Modern therapies have revolutionized the treatment of JIA as a whole [121], but the TMJ appears to have lagged behind [6]. Thus, IA therapy may remain the best option for some children. While we do not discount recent scholarship indicating the IACI may adversely impact the growth of the jaw [51], nor do we discount four decades of scholarship indicating that unopposed arthritis is harmful [42], and to date, corticosteroids are the only local therapy that have clearly shown to be of benefit in the management of TMJ arthritis. Future prospective research is indicated to evaluate alternative local approaches, as well as to understand the natural course among children with active inflammation, so that we can predict which children are likely to develop significant damage among those with active disease.

## Abbreviations

CBCT: Cone beam computed tomography
CE: Contrast enhancement
CT: Computed tomography
ER: Enhancement ratio
FOA: Functional orthodontic appliance
GBCA: Gadolinium-based contrast agent
HA: Hyaluronic acid
HBF: Heterotopic bone formation
IACI: Intraarticular corticosteroid injection
ICR: Internal condylar resorption
JIA: Juvenile idiopathic arthritis
LLLT: Low-level laser therapy
MIO: Maximal incisal opening
SI: Signal intensity
TH: Triamcinolone hexacetonide
TMJ: Temporomandibular joint
US: Ultrasound
